# Deep-time mass extinction helps understand the current biotic crisis

**DOI:** 10.1093/nsr/nwad322

**Published:** 2023-12-20

**Authors:** Shucheng Xie

**Affiliations:** State Key Laboratory of Biogeology and Environmental Geology, China University of Geosciences (Wuhan), China

Mass extinction, i.e. the above-background-level mortality of a large number of species within a short time period, attracts considerable attention from both scientists and the public. Earth has experienced at least five major biocrises (the so-called ‘Big Five mass extinctions’) since the advent of the first predators. A mountain of papers, including lots of special issues, have been published on this topic, leading to significant progress in documenting the patterns, processes and causes of these biocrises. To make a timely contribution to this opus of knowledge and, in particular, motivated by the desire to further understanding of current biospheric consequences, a Special Topic on mass extinction was organized here, including an interview, 3 review papers and 3 perspective papers.

The causes of mass extinction are one of the focuses of research in recent decades. Every mass extinction is known to have both a trigger (i.e. ultimate cause) and kill mechanism(s) (i.e. proximate cause(s)) [1]. The triggers can come from the Earth's interior (large igneous provinces (LIPs), tectono-oceanic changes), the Earth's exterior (bolide impacts, and some other astronomical drivers), and the biosphere itself. A common ultimate cause to explain all of the Big Five mass extinctions is unlikely, as current evidence favors LIP triggers for 2 biocrises (Permo-Triassic, end-Triassic), a bolide impact for 1 (end-Cretaceous), and bio-evolutionary events for 2 (Late Ordovician and Late Devonian). The proximate kill mechanisms are related to climatic and environmental changes (e.g. temperature, redox, pH, etc.) that can broadly be categorized as carbon-release and carbon-burial events. A particularly well-studied example is the Permo-Triassic biocrisis, which was characterized by a two-stage extinction accompanied by progressive warming in equatorial latitudes [2]. Two stages were also identified for the end-Ordovician biocrisis, occurring at the beginning and end of a rapid cooling that triggered a major ice age [3].

Key issues with regard to the response of modern ecosystems to climate change are ‘resilience’ and ‘recovery’ [4]. Mass extinctions represent natural experiments in which biotic systems have responded to major climato-environmental perturbations. Despite the catastrophic loss of species, post-extinction recoveries were generally regarded as rapid. The recovery from the Permo-Triassic mass extinction was temporally, geographically and ecologically patchy, providing an analog for prediction of the response of modern ecosystems to present-day climate stresses and a model for management of subsequent biospheric recovery [5].

Future investigations await the exploitation of advanced techniques. The adoption of high-resolution geochronology has demonstrated that both mass extinctions and subsequent recoveries could be more rapid than previously realized [4], and could help identify the global weirding associated with biocrises, which may be of particular relevance to present-day global change [6]. Application of analytical techniques to more effectively tap into microbial records will help us understand the role of microbes in major biocrises, whether serving as an accomplice or a savior [7]. Development of quantitative models for deep-time events will help us gain a more general understanding of resilience and recovery [4].

In conclusion, I would like to thank all authors, editorial staff, reviewers and everyone else who has contributed to the development and completion of this Special Topic.
